# Web-Based Module for the Collection of Electronic Patient-Reported Outcomes in People Living With HIV in Nouvelle Aquitaine, France: Usability Evaluation

**DOI:** 10.2196/15013

**Published:** 2019-12-18

**Authors:** Diana Barger, Olivier Leleux, Valérie Conte, Vincent Sapparrart, Marie Gapillout, Isabelle Crespel, Marie Erramouspe, Sandrine Delveaux, Linda Wittkop, François Dabis, Fabrice Bonnet

**Affiliations:** 1 University of Bordeaux ISPED, Inserm, Bordeaux Population Health Research Center Team MORPH3EUS, UMR 1219 Bordeaux France; 2 Centre de Recherche et Développement en Informatique Médicale University of Bordeaux Bordeaux France; 3 CHU de Bordeaux COREVIH Nouvelle Aquitaine Bordeaux France; 4 AIDES Nouvelle Aquitaine Bordeaux France; 5 CHU de Bordeaux Service d'information médicale, Pôle de sante publique Bordeaux France; 6 CHU de Bordeaux Service de médecine interne et maladie infectieuses Bordeaux France

**Keywords:** patient reported outcome measures, patient generated health data, quality of life

## Abstract

**Background:**

Patient-reported outcomes (PROs) can be of great value for both research and chronic disease management. We developed a new module of the ANRS CO3 Aquitaine cohort study’s Web-based data capture and visualization solution (APPEGE 2.0) for the collection of electronic PROs among people living with HIV cared for in Nouvelle Aquitaine, France.

**Objective:**

This study aimed to evaluate the usability of 2 successively developed prototypes of ARPEGE 2.0’s electronic PROs module before launching a pilot study, owing to the novelty of the proposed data collection method for our setting and specific characteristics of the target population.

**Methods:**

A total of 2 sequential rounds of empirical, task-based usability evaluations were conducted, involving 8 research staff and then 7 people living with HIV. Evaluators provided written feedback during round 1 and oral feedback during round 2. Evaluators who completed the full set of tasks responded to the System Usability Scale (SUS). We assessed changes in SUS scores between rounds and concluded usability testing when SUS scores reached a ceiling effect, defining good usability a priori as a usability score of 70.

**Results:**

Insights were generated regarding the visibility of system status and the match between the system and the real world that improved the module’s usability. Research staff evaluators reported mean SUS scores of 65 (SD 18.87) and patient evaluators reported mean SUS scores of 85 (SD 5.4; *P*=.032).

**Conclusions:**

Software modifications, informed by successive rounds of usability testing, resulted in sufficient gains in usability to undertake piloting. Insights generated during evaluations prompted us to find the appropriate balance between optimal security and ease of use.

**Trial Registration:**

ClinicalTrials.gov NCT03296202; https://clinicaltrials.gov/ct2/show/NCT03296202

**International Registered Report Identifier (IRRID):**

RR2-10.2196/10.2196/resprot.9439

## Introduction

HIV, once fatal, is now a manageable chronic illness [1]. In Western Europe, the majority of individuals who received a diagnosis of HIV are in care and on potent antiretroviral therapy, which prevents serious diseases both related and unrelated to AIDS [2]. The improved prognosis and the increased life expectancy of people living with HIV (PLWH) makes preserving health and ensuring good quality of life the cornerstone of their care [3-5]. One strategy to help providers respond to PLWH’s evolving needs and improve the quality and efficiency of their overall care is collecting and using patient-reported outcomes (PROs) [6].

PROs or “any report of the status of the patient’s health condition that comes directly from the patient, without interpretation of the patient’s response by a clinician or anyone else” [7] have been used extensively in clinical research [6]. PROs can be used at the population level for research and to improve the quality of care or at the individual level to support clinical decision making [8]. Their use may allow for more accurate symptom detection, better patient-provider communication, and improved outcomes [9]. Logistical, technical, and ideological barriers have nevertheless limited their use in routine care [10]. The adoption of electronic medical records coupled with the adaptation of paper questionnaires to computerized and internet-based formats may help overcome these barriers [10,11].

With evidence from the United States suggesting that the collection of PROs by using touchscreen-based information technology was both feasible and of value for both research and clinical HIV care [12-14], a prototype of an electronic PRO module linked to the ANRS CO3 Aquitaine cohort’s data capture and visualization system (ARPEGE 2.0) was developed in 2017 [15]. As the overall usefulness of interactive health care applications or their usability is likely to affect their acceptability and adoption, usability evaluations of 2 successively developed prototypes of the ARPEGE 2.0 solution were conducted in preparation for a pilot study [15].

## Methods

This formative research study took place in Bordeaux, France, at the Inserm UMR 1219-Bordeaux Population Health Research Centre and the St André Bordeaux University Hospital. It was designed as part of the ANRS CO3 Aquitaine study, an open, prospective hospital-based cohort of PLWH in care in 13 clinics in southwestern France. A local institutional review board approved the study’s protocol (Comité de Protection de Personnes Sud-Ouest et Outre-Mer III) on September 18, 2017.

### Description of the Electronic Patient-Reported Outcome Module Powered by ARPEGE 2.0

ARPEGE 1.0 is a proprietary, secure, electronic case report form developed in Microsoft ASP.NET (WebForm). Data are stored within a Microsoft SQL Server 2014–based data management system. The ANRS CO3 Aquitaine cohort relies on ARPEGE 1.0 for data capture. Clinical data, extracted from both medical records and laboratory data, derived from the hospital’s laboratory information management systems, have been collected systematically since 1987 and electronically via ARPEGE 1.0 since 2013 with the support of Clinical Research Associates. ARPEGE 2.0 is a generic Web-based data capture and visualization system also developed in Microsoft ASP.NET (WebForm). ARPEGE 2.0 has enabled the creation of the module for the collection of electronic PROs in routine care for observational research and, ultimately, clinical care.

The content of ARPEGE 2.0’s initial electronic PRO module is based on current treatment guidelines for people being treated for HIV and associated comorbidities [16]. Prototyping was carried out over 2017 with the support and regular feedback from a working group comprising research staff, local stakeholders, and end users (clinicians and patient representatives). The questionnaires were evaluated individually according to their psychometric properties, administration method, and length. The following areas are covered by the electronic PRO module: socioeconomic status and individual social and material deprivation [17], multidimensional quality of life (WHOQOL-HIV BREF) [18], treatment burden (Treatment Burden Questionnaire) [19], physical activity (the Short Version of the International Physical Activity Questionnaire), alcohol use and screening for at-risk drinking behavior (Alcohol Use Disorders Identification Test Consumption, Fast Alcohol Consumption Evaluation) [20], tobacco and nicotine use and screening for tobacco dependency (Fagerström), cannabis (Cannabis Abuse Screening Test) and drug use, and, finally, depression (Patient Health Questionnaire) [21].

Conditional branching was used where appropriate. The module also allows patients to report any other treatment-related issues in a free text field. Where applicable, the International Society for Pharmacoeconomics and Outcomes Research ePRO Task Force’s recommendations on adapting paper-based instruments were followed, ensuring that data produced are equivalent or superior to those generated from paper-based administration methods [22].

### Recruitment

Nielsen’s recommendations that favor conducting several iterative studies, each with a small number of participants, were adopted [23]. In round 1 (May 2018), evaluators were employees of the Inserm UMR 1219 Bordeaux Population Health Research Center or affiliated with the project, referred to herein as *research staff*. In round 2 (June 2018), a convenience sample of PLWH being cared for at the St André Bordeaux University Hospital was identified by clinical staff either before or during their routine visit.

### Procedure

The evaluation procedure differed between round 1 and round 2. However, for both rounds, oral consent was obtained. It was then explained that each study participant (evaluator) would be provided with a unique identifier, which would allow him/her to create a personal account and access the questionnaires. Evaluators were shown the study-specific brochure where the number would be written on a detachable coupon (Multimedia Appendix 1). They were asked to complete 5 tasks: (1) navigate between pages on the publicly available website and locate key information, (2) create a user account, (3) confirm their account, (4) initiate the electronic PRO assessment, and (5) complete the electronic PRO assessment. Whether or not each task was completed with ease, assistance or not was monitored, and a score of 2 to 0 was attributed (2=the task was completed with ease and 0=it was not completed). The highest possible score was therefore 10, and the lowest score was 0. Neither round 1 nor round 2 evaluators were compensated.

In round 1, research staff were provided with instructions detailing the background of the study and how it would be implemented in a clinical setting. Evaluators were given a link to a staging version of the electronic PRO module. They were asked to complete the previously described tasks. They then responded to an Web-based questionnaire that included the System Usability Scale (SUS), a widely used robust tool for measuring usability. It consists of 10 items with 5 response options, from strongly agree to strongly disagree [24,25]. Evaluators provided written feedback in an open text field and by email.

In round 2, patients participated in one-on-one testing sessions, lasting between 1 and 2 hours, with a researcher in a dedicated, private space at the hospital (June 2018). The researcher based each session on a standardized qualitative interview guide. A personal computer (Mac Book Air) with access to the staging site was provided to complete the study tasks. Patient evaluators were also allowed to complete the questionnaire on their personal smartphones, matching how the electronic PRO module might be accessed in routine care. Evaluators were instructed to use the *think aloud* method, in which users are asked to verbalize all thoughts as they interact with the system while carrying out tasks. Subsequently, those who completed all tasks responded orally to the SUS and provided open-ended feedback [24]. All sessions were audio recorded, and field notes were taken.

### Analysis

Task completion and SUS scores were calculated for each evaluator, and means and standard deviations were calculated for each round. We performed a *t* test assuming unequal variance to determine if each round of testing produced significant difference in the mean SUS scores. A priori, we defined *success* in usability when the SUS score reached a ceiling effect, with a minimum score of 70—generally accepted as a cut-off for *good* usability [26].

Qualitative analysis included review of written feedback, audio recording–enhanced field notes, and responses to open-ended questions. We performed thematic content analysis on written feedback and audio recording–enhanced field notes, abstracting and compiling emerging themes from each round of testing. These are reported according to Nielsen’s usability heuristic categories [27].

## Results

### Overview

Table 1 presents evaluators’ characteristics and mean task completion scores for rounds 1 and 2. The majority of round 1 evaluators were women (7/8). They reported using computers either regularly (5/8) or often (3/8). In all, 5 out of 7 round 2 evaluators were men. A total of 3 reported using a computer regularly, 3 often, and 1 never. Overall, mean task completion scores were 7.8 (out of 10) in round 1 and 7.1 in round 2. In round 1, 7 evaluators completed all tasks compared with 4 out of 7 in round 2. Task completion was hampered owing to 2 evaluators being locked out of their accounts and 1 evaluator being unable to complete tasks owing to poor eyesight. This evaluator was attributed 0 on all tasks.

The usability insights uncovered during the 2 rounds of usability evaluations together with the solutions adopted are presented in Table 2.

**Table 1:** Evaluator characteristics and task scores.

Evaluator characteristics			Task 1—information found	Task 2—account created	Task 3—account confirmed	Task 4—PRO^a^ assessment initiated	Task 5—PRO assessment completed	Total
**Round 1 (N=8)**			2.0	1.1	1.4	1.5	1.8	7.8
	**Male (n=1)**		2.0	0.0	0.0	0.0	0.0	2.0
		30-40	2.0	0.0	0.0	0.0	0.0	2.0
	**Female (n=7)**		2.0	1.3	1.6	1.7	2.0	8.6
		<30	2.0	1.5	1.5	1.5	2.0	8.5
		30-40	2.0	1.3	1.3	1.7	2.0	8.3
		41-50	2.0	1.0	2.0	2.0	2.0	9.0
		>50	2.0	1.0	2.0	2.0	2.0	9.0
**Round 2 (N=7)**			1.7	1.1	1.4	1.7	1.1	7.1
	**Male (n=5)**		1.6	1.2	1.6	1.6	1.6	7.6
		<30	2.0	1.5	2.0	2.0	2.0	9.5
		30-40	2.0	2.0	2.0	2.0	2.0	10.0
		>50	1.0	0.5	1.0	1.0	1.0	4.5
	**Female (n=2)**		2.0	1.0	1.0	2.0	0.0	6.0
		>50	2.0	1.0	1.0	2.0	0.0	6.0

^a^PRO: patient-reported outcome.

**Table 2:** Usability insights per round and solution adopted according to Nielsen’s usability heuristics.

Usability categories	Round 1—research staff	Round 2—patients	Solution
Visibility of system status	Login procedure was confusing owing to the complexity of password, requiring 2 symbols	Challenges adhering to password requirements for certain patients	Password requirements were spelled out for users in bold. A password visualization button was also added to the password field to allow users to ensure that passwords created matched before registering their account
	—^a^	Unclear whether the QuAliV number (required for creating the account) is case sensitive	Information incorporated into the presentation of the study to participants
	Validation of questionnaire unclear	—	Information buttons added to the home page of the electronic PRO^b^ module instructing users on how the questionnaires functioned and reminding them to *submit* their completed questionnaires. The button was also relabeled to make its functionality clearer
Match between system and the real world	Date picker was in English and began in 2018, requiring users to click to go back in time	—	The date picker was replaced with a French version. It allowed users to type in their birth dates without using the calendar
	—	Nonmutually exclusive modalities or response missing	Minor modifications made to question modalities to ensure clarity
	—	Issues stemming from the translation of questionnaire from English to French	Further cognitive debriefing with native speakers to identify the best translation of the item in question
	—	Difficulties understanding the meaning of certain questions	Less formal language substituted where possible and examples given to facilitate the comprehension of certain questions
	—	Confirmation of account on one’s smartphone (email) resulted in being locked out of one’s account on another device	Automatic connection to the site after creating one’s account deleted (temporarily) to avoid users locking themselves out of their account. Users must reenter their username and password
User control and freedom	Need for returning back to last page completed in the questionnaire	—	The user is now redirected back to the most recent page completed within each questionnaire. Scrolling from one page of a questionnaire to another automatically saves entered data
	Radio button could not be unclicked or erased	—	A refresh button was added to each item to allow users to erase their responses and therefore leave items unanswered
	Questionnaire opens in a pop-up window whose size cannot be modified	—	Double checked to ensure that text could be easily read in each window
	—	Unclear whether users had to provide first and last name	We added text indicating that typing one’s first and last name was optional
Consistency and standards	Format of certain questions was noted as being inconsistent between questionnaires. Yes/No questions appeared in a table format as soon as they used the same response thesaurus	—	Minor improvements in formatting were made where possible. Further development required to accommodate this change in the longer run
	Typos in certain questions were identified	—	—
Error prevention	Aberrant response possible for certain free text fields	—	Stricter constraints added
	The password required was complex. Instructions on password requirements were missing from the account creation page	—	Instructions on password requirements added
	Need to clarify units in free text fields		Units added in gray in each text field
	Need to indicate which questions were mandatory in the questionnaire. Need to indicate when multiple answers could be given	—	An asterisk was added to indicate which questions were mandatory. The user is sent back to mandatory questions before being allowed to progress in the questionnaire. These questions were marked in red to indicate that they were mandatory
Recognition rather than recall	Automatic logout obligations meant that users could not reconnect to their accounts for 20 min if they left the page, resulting in certain evaluators being locked out of their account	—	Error message added to the module explaining that users would be able to reaccess their accounts after 20 min
Flexibility and efficiency of use	Errors encountered with the progress bar depending on responses to questions	—	Questionnaires are programmed to open successively
	Errors on certain Web browsers	—	Further trouble shooting using full array of browsers and devices
Aesthetic and minimalist design	Methods for completing a visual analog scale unclear as definition of extreme values was missing, and a *not applicable* box was not included	—	An 11-point radio button scale was proposed as a temporary solution
	The IPAQ^c^ questionnaire was difficult to read on the pop-up screen	—	Alternative formatting used to improve readability
Help users with errors	Need to flag missed items	—	Progression bar for each questionnaire goes from orange to green as soon as all nonconditional questions are answered. Users are directed to unanswered obligatory questions upon attempting to go on to the next page of the questionnaire
Help and documentation	Information missing from different links (contact and preferences)	—	—
	Print button of informed nonopposition did not function correctly	—	—

^a^Not applicable.

^b^PRO: patient-reported outcome.

^c^IPAQ: International Physical Activity Questionnaire.

### What Worked

The first task involved navigating the external website that patients would access from home, unassisted, to create their account. Users found the information provided on the external website quickly and found its structure clear. All users quickly understood how the attributed unique identifier would be used to create their personal account. Once users had created their account, efforts to guide him/her through electronic PROs by having each questionnaire open one after the other appeared to work well. The use of stoplight-style color coding and a progress bar allowed users to see if they had missed a question and helped them recognize, diagnose, and recover from errors seamlessly. The order of the PROs was received positively by users and therefore remained unchanged between prototype versions.

### What Did Not Work

**Figure 1:**
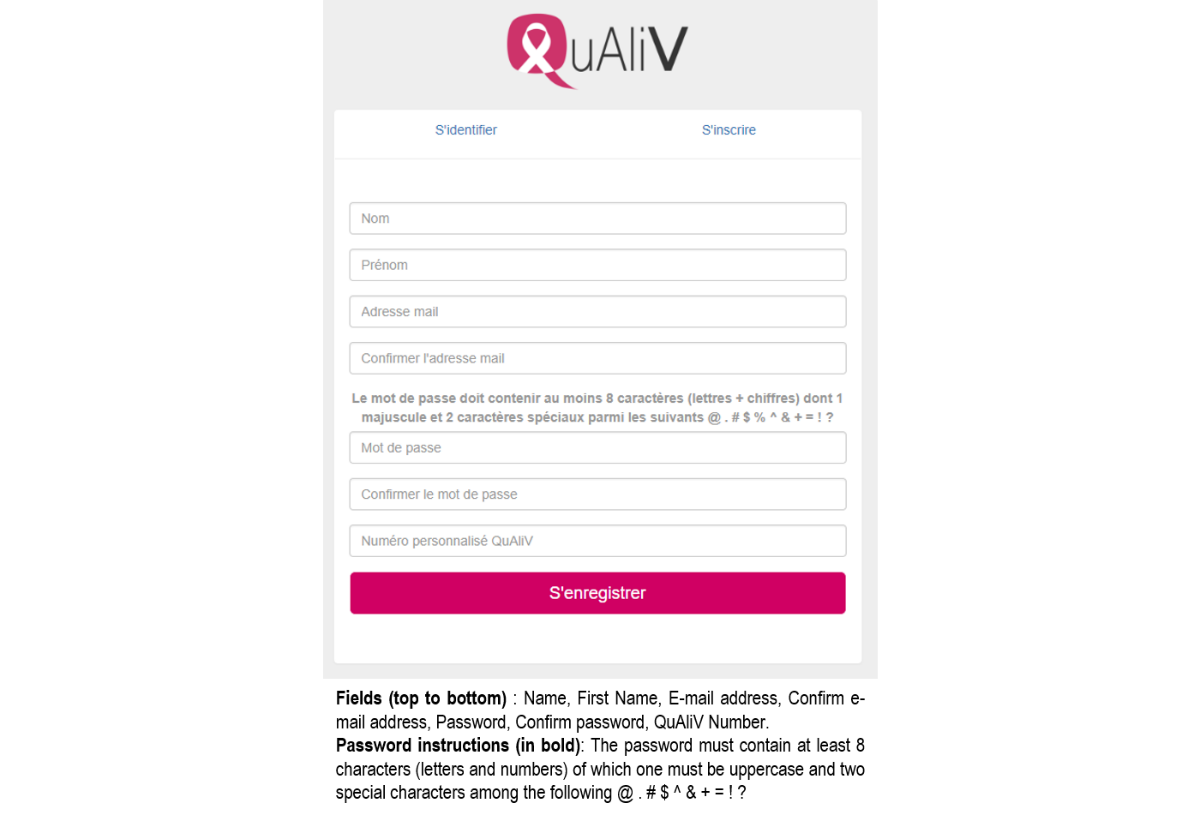
Initial log-in page (round 1).

**Figure 2:**
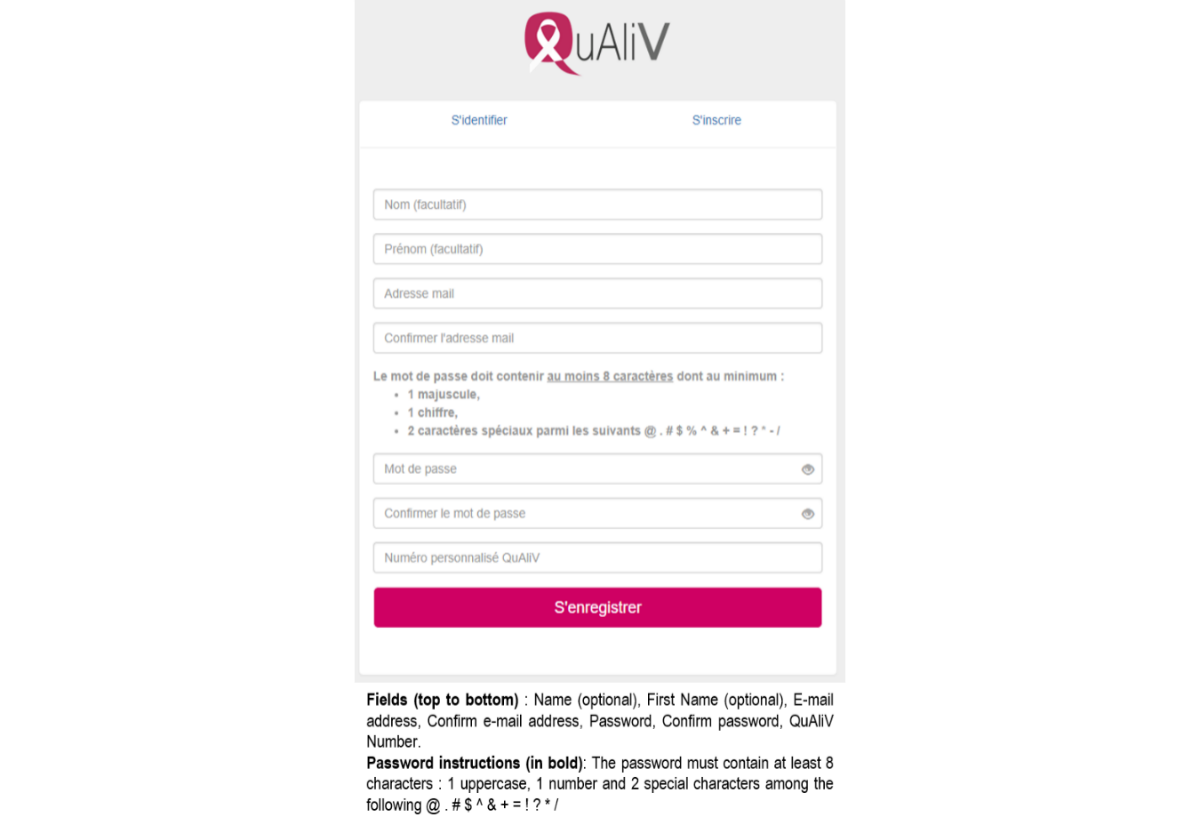
Revised log-in page (round 2).

The account creation task was the most challenging for users. One of the issues identified was the complexity of the password requirements. The password had to be entered twice and contain at least 8 alphanumeric characters, including 2 special characters and a capital letter (Figure 1). Many evaluators, both research staff and patients, attempted this step more than once. We clarified the password requirements and ensured that error messages were informative regarding the system status, and we made it possible to visualize the password after round 1 (Figure 2). As errors still occurred, we added additional error prevention features. The password is validated as the user types as opposed to the user receiving an error message upon clicking *register* (Multimedia Appendix 2).

A login problem, also detected during the second round of usability testing, was being *locked out* of one’s account accidentally. This issue arose from a security measure included in the electronic PRO module’s design. Users were logged out automatically after a period of 20 min of inactivity. If users accidently left the page without logging out of their accounts, they could no longer log back in owing to the Bordeaux University servers’ restrictions. If the user attempted to return to their account, they received an error message indicating that they were already connected. This issue could not be resolved without completely relaxing the automatic logout timeframe (shortening it). We therefore modified the error message indicating that the user could access their account again in 20 min.

### System Usability Scale Scores

In round 1, experts reported mean SUS scores of 65 (SD 18.87), and patients, in round 2, reported mean SUS scores of 85 (SD 5.4) (*P*=.032).

## Discussion

### Principal Findings

Iterative usability evaluations of 2 successively developed prototypes allowed us to see how easy our electronic PRO module was to use and identify when and where users encountered problems or experienced confusion. We were able to improve the module’s usability markedly, specifically the visibility of system status and the match between the system and the real world, and take into account the specific needs of our patient population (their level of computer literacy and age) and the specificities of our clinical setting. Finally, we were pushed to find the appropriate balance between optimal security and ease of use.

Unlike PRO collection methods employed in clinics in the United States [12-14], where patients complete an electronic PRO assessment by using touchscreen information technology with the assistance of a research assistant/administrator at clinics, we aimed to design a Web-based *Bring Your Own Device* solution. We therefore assumed that the majority of users would have access to a smartphone or personal computer with a reliable internet connection. The proposed solution, developed in-house, had to work well enough to allow a group of users, with varying levels of computer familiarity, to use it with little to no assistance.

### Strengths and Limitations

Some caveats should be considered in the interpretation of our results. We conducted the first round of usability testing in a sample of research staff who may not fully represent end users. This strategy, recognized as an easy way of catching obvious usability issues, resulted in high-quality, detail-oriented, and exhaustive feedback, allowing for a number of basic usability problems to be resolved before evaluations with patients. Most evaluators were comfortable using computers and the internet. They may not fully reflect the diversity of the cohort of PLWH in the region. More purposeful sampling of evaluators with lower computer literacy may have resulted in the detection of additional usability insights.

In round 2, we used the *think aloud* method. This method has been known to slow the thought process and increase mindfulness, which might prevent errors that might have normally occurred [28]. However, when evaluators are asked to perform simple tasks, the method has been shown to have no effect on user performance [29]. We opted for this method as the tasks were not considered complex.

### Conclusions

Nevertheless, software modifications, informed by successive rounds of usability testing, resulted in sufficient gains in usability to undertake piloting.

## Appendix

Multimedia Appendix 1 Patient brochure with unique identifier.

Multimedia Appendix 2 Login page with password verification.

## Abbreviations

PLWH: people living with HIV
PRO: patient-reported outcome
SUS: System Usability Scale
