# Supplementary figures and images for: Web-Based Module for the Collection of Electronic Patient-Reported Outcomes in People Living With HIV in Nouvelle Aquitaine, France: Usability Evaluation
Source: JMIR Form Res. 2019 Dec 18;3(4):e15013. doi: 10.2196/15013 (PMC6939280; doi:10.2196/15013)

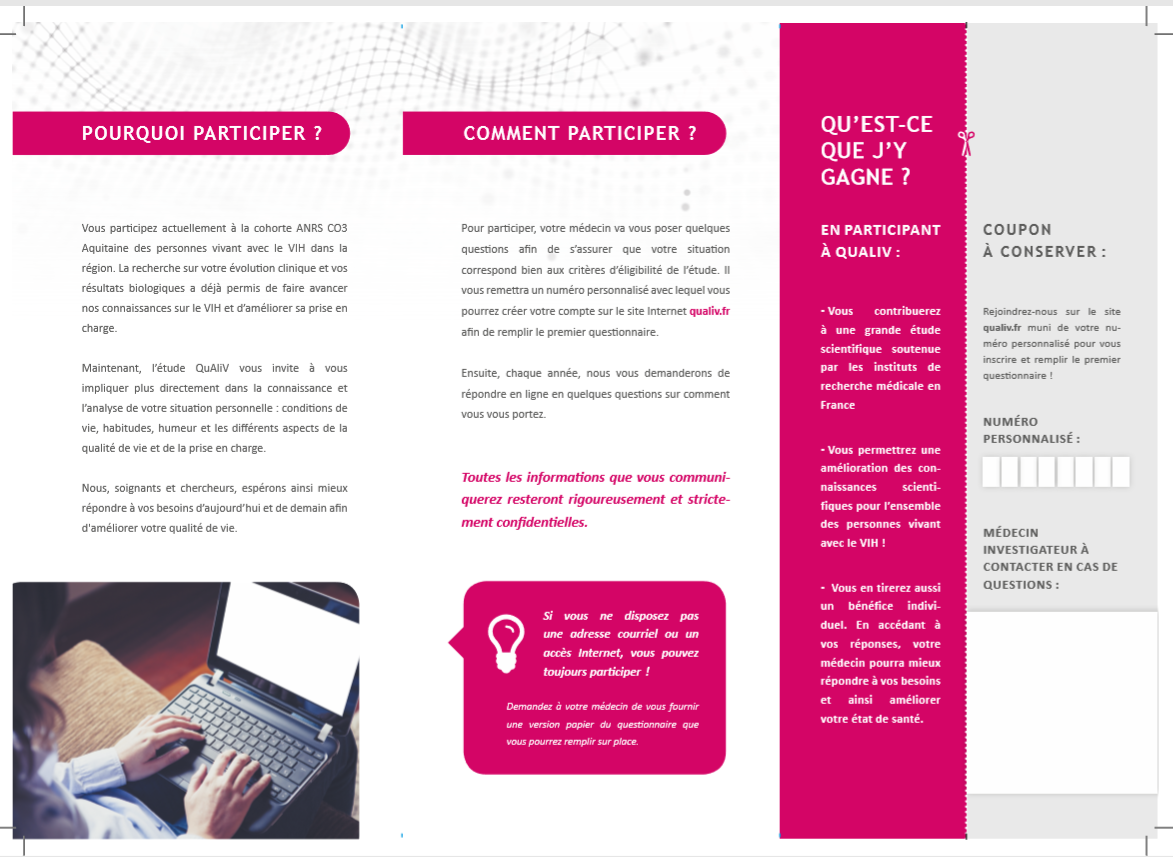

Supplement: Multimedia Appendix 1 [file formative_v3i4e15013_app1.png]

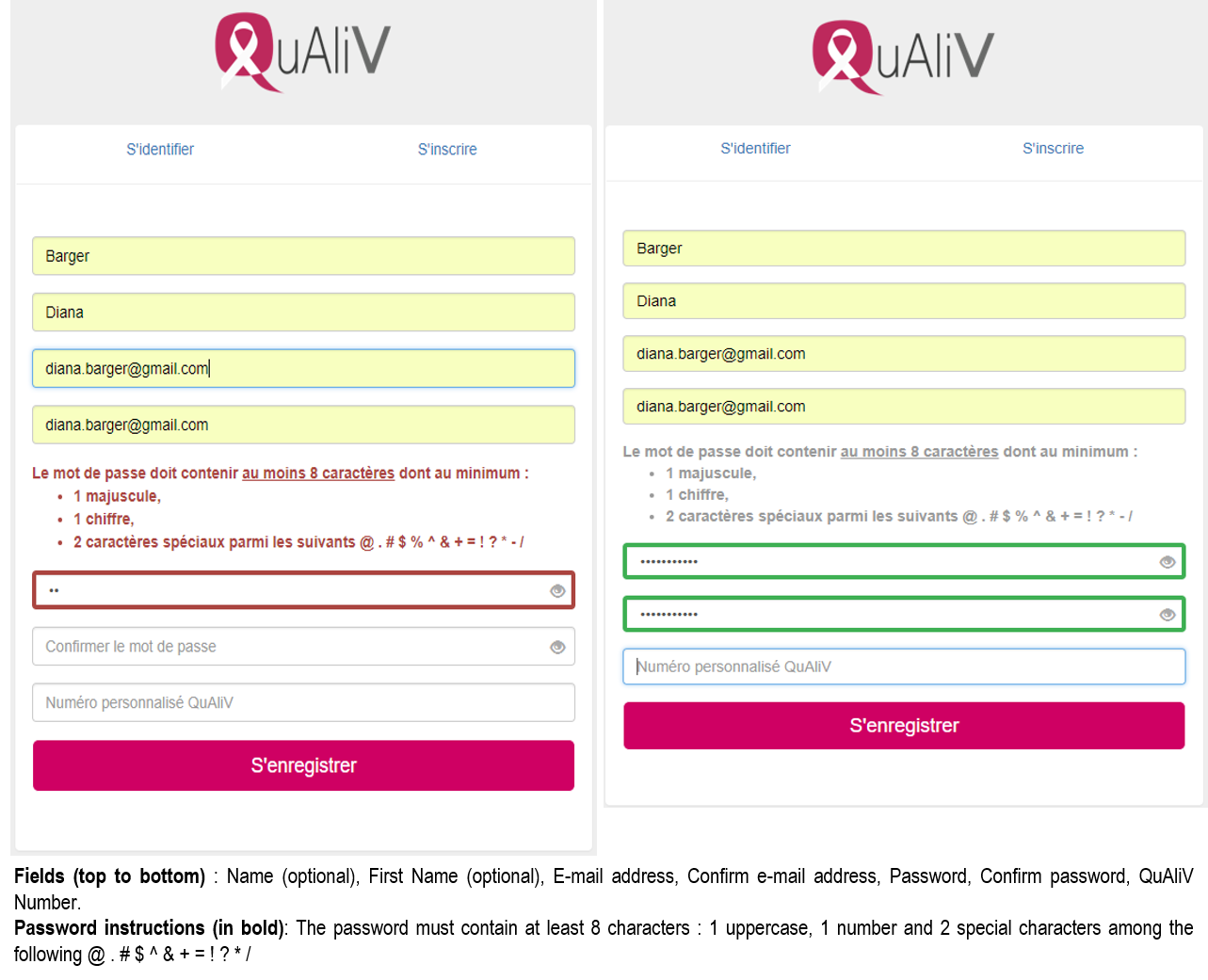

Supplement: Multimedia Appendix 2 [file formative_v3i4e15013_app2.png]
